# Supplementary material for: Effects of Two Different Straw Pellets on Yak Growth Performance and Ruminal Microbiota during Cold Season
Source: Animals (Basel). 2023 Jan 17;13(3):335. doi: 10.3390/ani13030335 (PMC9913257; doi:10.3390/ani13030335)
Supplement: Supplementary file 1 [file animals-13-00335-s001.zip › animals-2130859-supplementary.pdf]

Table S1: The composition of the premix

Item	VA	VD3	VE	Ca	P	Cu	Fe	Zn
content/(kg)	90 000 IU	30 000 IU	350 IU	120 g	20 g	160 mg	1200 mg	2000 mg
